# Type D personality and fear of progression among patients with first-ever stroke: the serial mediation role of perceived social support and intolerance of uncertainty

**DOI:** 10.3389/fpsyg.2025.1628451

**Published:** 2025-08-05

**Authors:** Xiaoping Yang, Lijun Wang, Xiaohui Liu, Miaomiao Chen, Yingjie Zheng, Shailing Ma, Jialin Yuan, Huijuan Wang

**Affiliations:** ^1^General Hospital of Ningxia Medical University, Yinchuan, China; ^2^School of Nursing, Ningxia Medical University, Yinchuan, China

**Keywords:** stroke, type D personality, perceived social support, intolerance of uncertainty, fear of progression, serial mediation role

## Abstract

**Objective:**

Fear of progression (FoP) significantly impacts multidimensional health outcomes in stroke patients. Although Type D personality predicts FoP, mechanisms underlying the association between Type D personality and FoP remain poorly understood. This study aimed to investigate the impact of Type D personality on FoP in first-ever stroke patients and the serial mediation role of perceived social support (PSS) and intolerance of uncertainty (IU).

**Methods:**

This cross-sectional study employed convenience sampling to recruit 300 patients with first-ever stroke (228 males and 72 females; mean age 59.52 ± 12.72 years) from two tertiary hospitals in Yinchuan, China. Participants completed the following scales: the General Information Questionnaire, the 14-item Type D scale, the Multidimensional Scale of Perceived Social Support, the Intolerance of Uncertainty Scale-12, and the Fear of Progression Questionnaire-Short Form. SPSS 24.0 was used for Harman’s single-factor test, descriptive statistics, Pearson correlation analysis, and regression analysis, with serial mediation role examined using the PROCESS macro v3.5.

**Results:**

Our results showed that: (1) the direct effect of Type D personality on FoP was significant. (2) PSS did not independently mediate the Type D personality-FoP relationship. (3) IU significantly mediated the Type D personality-FoP relationship. (4) PSS and IU demonstrated the significant serial mediation role between Type D personality and FoP.

**Conclusion:**

Type D personality exerted a direct effect on FoP among first-ever stroke patients. The serial mediation model demonstrated that enhancing PSS could reduce IU, thereby alleviating FoP. Interventions targeting PSS enhancement and IU reduction constitute a promising approach to mitigate FoP in these patients, despite Type D traits’ inherent stability.

## 1 Introduction

According to the Global Burden of Disease Study 2024, stroke ranks as the third leading cause of global mortality (GBD 2021 Stroke Risk Factor Collaborators, 2024), and is a major contributor to death and disability among adults in China (Liu et al., 2025). Clinically, stroke manifests with complex and diverse symptoms, necessitates prolonged recovery, and exhibits high recurrence rates. Notably, 70–80% of survivors experience residual disabilities post-treatment (Yang et al., 2025). These enduring physical and psychological burdens, compounded by limited patient understanding and uncertainty regarding treatment and prognosis, foster a prevailing fear of progression among stroke patients (Yang et al., 2025).

Fear of Progression (FoP) refers to a patient’s fear of the biopsychosocial consequences associated with disease progression or the fear of disease recurrence (Dankert et al., 2003). As one of the most prevalent unmet psychological needs in stroke patients (Townend et al., 2006), FoP is frequently conceptualized as a ‘Damoclean threat’—an evocative metaphor emphasizing the constant vigilance and apprehension maintained by patients with stroke toward potential disease progression (Sharpe et al., 2023). While moderate FoP may serve as an adaptive response that promotes health behaviors (Ocalewski et al., 2021), excessive FoP can trigger pathological health anxiety, impair quality of life, and potentially elevate cerebrovascular event risk (Faezi et al., 2017; Simonelli et al., 2017). Therefore, FoP should be considered in the management of stroke patients.

### 1.1 Type D personality and FoP

Type D personality is a stable trait characterized by persistent negative affectivity and social inhibition (Denollet, 2005). Patients exhibiting this trait typically display pessimistic attitudes toward clinical prognosis and express frequent concerns about disease progression (Mols et al., 2012). Yin et al. (2023) reported a high prevalence of Type D personality among patients with stroke and identified it as a robust predictor of post-stroke depression. Furthermore, evidence from patients with lung cancer revealed that Type D personality significantly predicts elevated FoP (Lv, 2017). Similarly, a study of patients undergoing valvular heart surgery confirmed Type D personality as a risk factor for severe FoP (Wu et al., 2025). However, the relationship between Type D personality and FoP in first-ever stroke patients remains underexplored. Therefore, we hypothesize:

*H1:* Type D personality in patients with first-ever stroke positively predicts FoP.

### 1.2 The mediating role of perceived social support (PSS) in the relationship between Type D personality and FoP

Perceived social support (PSS) refers to an individual’s subjective appraisal of the availability and adequacy of resources provided by their social networks (Paykani et al., 2020). PSS represents a critical determinant for maintaining mental health (Tonsing et al., 2012). Substantial evidence indicates that Type D personality is associated with impaired PSS, as demonstrated by significantly lower PSS levels in Type D compared to non-Type D patients with coronary artery disease (Ginting et al., 2016). Furthermore, O'Riordan et al. (2020) identified reduced PSS as a mediator between a Type D personality and cardiovascular reactivity to acute stress. Crucially, lower PSS predicts heightened FoP in diverse clinical populations. Guan et al. (2024) documented a negative correlation between PSS and FoP in patients with stroke, while a study on patients with primary brain tumors confirmed that lower PSS significantly predicts increased FoP (Du et al., 2024). These findings suggest that PSS may significantly mediate the relationship between Type D personality and FoP. Consequently, we hypothesize:

*H2:* PSS mediates the relationship between Type D personality and FoP in first-ever stroke patients.

### 1.3 The mediating role of intolerance of uncertainty (IU) in the relationship between Type D personality and FoP

Intolerance of uncertainty (IU) is the tendency to react negatively to uncertain situations (Näsling et al., 2024). Individuals with high IU tend to catastrophically interpret uncertain future events, viewing uncertainty as highly threatening and unacceptable (Malivoire et al., 2019). Multiple studies consistently demonstrate that personality traits significantly predict IU levels (Bongelli et al., 2021; Zhang et al., 2022; Kumar et al., 2025). Given that stroke progression and outcomes are inherently uncertain, affected patients inevitably face substantial disease-related uncertainty (Salter et al., 2008). This challenge is particularly pronounced among individuals with a Type D personality, who exhibit markedly diminished tolerance for ambiguity (Grynberg et al., 2012). Critically, heightened IU predicts increased FoP in clinical populations. Curran et al. (2020) demonstrated the predictive role of IU for FoP in patients with cancer, while Shen et al. (2024) established a strong positive correlation wherein lower IU levels correspond to reduced FoP. These findings imply that the relationship between Type D personality and FoP may be significantly mediated by IU. Therefore, we hypothesize:

*H3:* IU mediates the relationship between Type D personality and FoP in first-ever stroke patients.

### 1.4 The serial mediation role of PSS and IU

According to the Stress and Coping Theory (Folkman et al., 1986), individuals’ coping resources can affect their coping responses. In response to a stressor, the availability of coping resources can affect stress appraisal, coping responses, and health outcomes. In this study, stroke is considered a significant health-related stressor, while PSS constitutes a critical coping resource. Patients with Type D personality tend to perceive insufficient social support, and this deficit in coping resources exacerbates their IU, thereby promoting maladaptive coping responses—such as FoP in first-ever stroke patients. Previous studies have also confirmed that PSS has a negative impact on IU. For instance, a survey by Ne'eman-Haviv et al. (2025) revealed that PSS was negatively associated with IU. This suggests that PSS and IU may serve as serial mediators linking Type D personality to FoP in first-ever stroke patients. Based on this, we propose the fourth hypothesis:

*H4:* Type D personality influences FoP through the serial mediation role of PSS and IU among first-ever stroke patients.

**Figure 1:**
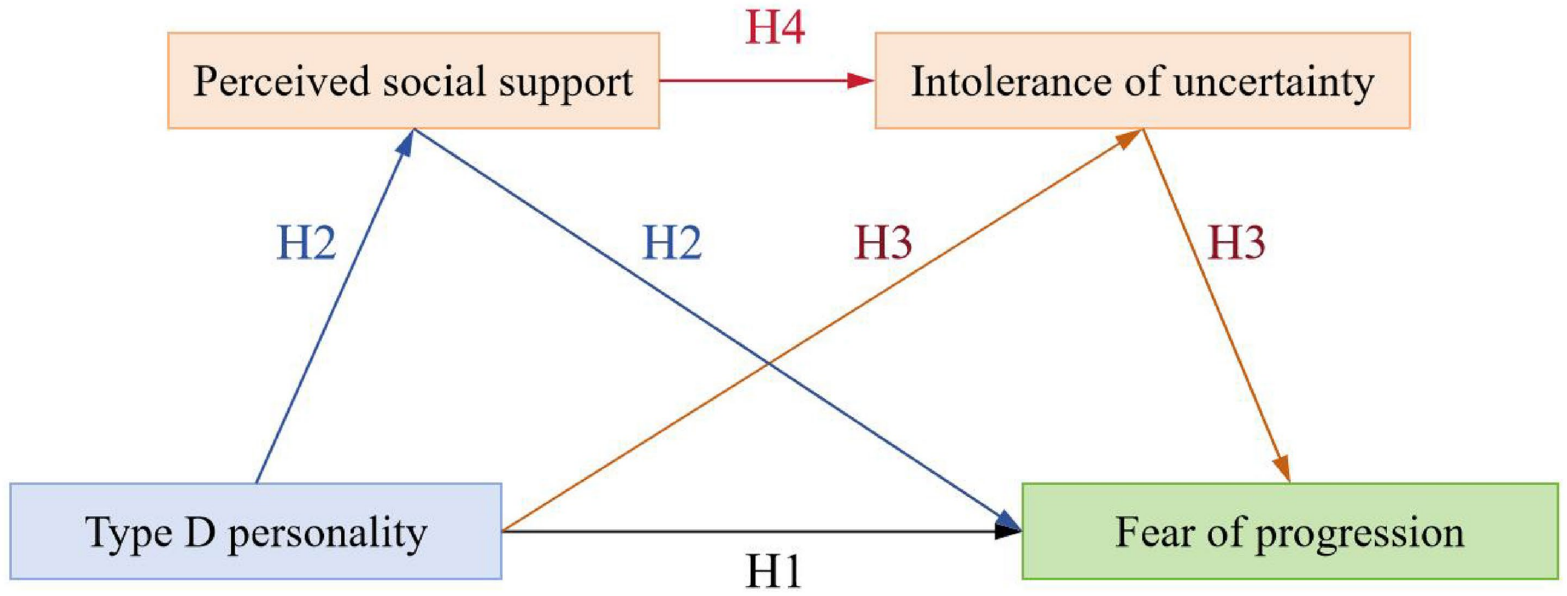
Hypothetical model.

Consequently, this study aimed to investigate the impact of Type D personality on FoP among first-ever stroke patients and the serial mediation role of PSS and IU in this relationship. As illustrated in Figure 1, this study proposed a hypothesized model to examine the underlying mechanisms through which Type D personality influences FoP.

## 2 Materials and methods

### 2.1 Ethics statement

The research protocol involving human participants was approved by the Institutional Review Board of Ningxia Medical University (Approval No. 2023-044). All patients participated voluntarily, and informed consent was obtained after explanation of the study purpose. We adhered to the Declaration of Helsinki and ethical principles throughout the study.

### 2.2 Participants

This cross-sectional study recruited participants from neurology inpatient departments at two tertiary hospitals in Yinchuan City, Ningxia, China, between May and September 2023. The inclusion criteria comprised the following: (a) adult patients who were stroke survivors meeting cerebrovascular disease diagnostic criteria, with ischemic stroke or hemorrhagic stroke confirmed by neuroimaging modalities (computed tomography [CT] or magnetic resonance imaging [MRI]); (b) aged ≥18 years; (c) had an illness duration of ≤2 weeks from onset; (d) experienced a first-ever stroke; and (e) provided written informed consent. The exclusion criteria encompassed patients who: (a) exhibited severe linguistic barriers or cognitive impairments, precluding effective communication and scale assessment; (b) had documented psychiatric disorders; and (c) presented with other severe systemic diseases, such as heart failure and malignant tumors.

### 2.3 Sample size and data collection

Based on cross-sectional study sample size requirements and Kendall’s criterion (Guan et al., 2024), the minimum sample size should be 5–10 times the number of study variables. With 20 variables included in this research, 100–200 participants were required. Accounting for potential invalid responses (estimated at 10–20% of questionnaires), we recruited 300 participants for this investigation. *Post hoc* statistical power analysis was conducted using G*Power 3.1™. The analysis demonstrated a test power of 99.9%, confirming that the sample size of 300 provides robust capability to detect the hypothesized effects (Cohen, 1992).

Researchers received standardized training in data collection methods prior to the formal investigation. Data were collected via face-to-face interviews. First, the research team explained the study’s purpose to eligible participants; those willing to participate provided written informed consent. During data collection, researchers delivered standardized instructions for the assessment tools, after which participants completed questionnaires independently. Researchers remained available to clarify questions or address participant concerns. For individuals unable to self-complete questionnaires, researchers administered items orally and recorded responses. The average completion time was approximately 20–30 min per questionnaire. All questionnaires underwent immediate quality checks to identify and rectify missing data.

### 2.4 Measures

#### 2.4.1 General information

We collected socio-demographic and clinical data using a General Information Questionnaire. These included variables such as gender, age group, education level, hospitalization costs, urban–rural distribution, employment status, marital status, Parental status, family financial situation, family history of stroke, types of stroke, complications, functional impairment, the number of other chronic diseases, daily living activities [evaluated using the Barthel Index (Oliveira et al., 2023)], and patients’ understanding of stroke-related knowledge.

#### 2.4.2 Type D personality

The 14-item Type D scale (DS 14), developed by Denollet (2005), was used to detect an individual’s Type D personality traits. The scale contains 14 items and two subscales; seven items measure the Negative Affectivity dimension (NA, covering dysphoria, worry, and irritability), while seven items evaluate the Social Inhibition dimension (SI, covering discomfort in social interactions, reticence, and lack of social poise). The scale was scored on a 5-point Likert scale varying from 0 = “False” to 4 = “True.” Scores on both NA and SI range from 0 to 28. An individual was categorized as having a Type D personality if the total score was 10 or higher for the NA and SI. However, a previous study suggested that a Type D personality may be more accurately represented as a dimensional rather than a categorical construct (Ferguson et al., 2009). The Cronbach’s *α* coefficient of the DS 14 in this study was 0.913.

#### 2.4.3 Perceived social support (PSS)

The Multidimensional Scale of Perceived Social Support (MSPSS), developed by Zimet et al. (1990), was used to measure PSS. The scale comprises 12 items across three subscales: Family Support, Friend Support, and Other Supports, which, respectively, reflect an individual’s perception of support from family members, friends, and other sources (e.g., leaders, colleagues, and relatives). Responses are rated on a seven-point Likert scale (1 = “strongly disagree” to 7 = “strongly agree”), with total scores ranging from 12 to 84. Higher scores indicate greater PSS. The Cronbach’s *α* coefficient of the MSPSS in this study was 0.927.

#### 2.4.4 Intolerance of uncertainty (IU)

The Intolerance of Uncertainty Scale-12 (IUS-12) was used to assess each participant’s tolerance for the occurrence of future uncertainties. This scale was developed by Carleton et al. (2007). The scale includes 12 items and two subscales, namely, Prospective Anxiety (involves fear and anxiety based on future events) and Inhibitory Anxiety (describes uncertainty inhibiting action or experience). The IUS-12 uses a 5-point Likert scale without reverse scoring, meaning higher scores indicate greater IU. In this study, the Cronbach’s *α* coefficient for the IUS-12 was 0.881.

#### 2.4.5 Fear of progression (FoP)

The Fear of Progression-Questionnaire-Short Form (FoP-Q-SF), developed by Mehnert et al. (2006), was used to measure participant’s FoP. The scale comprises two subscales: Physical Health (referring to patients’ concerns about their own physical health status, including fears of symptom worsening, functional decline, and treatment uncertainty) and Social-Family (reflecting patients’ fears about the disease’s impact on their social functions and family roles, covering concerns about social interaction difficulties, family responsibility burdens, work capability impairment, and relationship strain). The scale includes 12 items rated on a 5-level Likert scale, with total scores ranging from 12 to 60, with higher scores indicating higher levels of FoP. The Cronbach’s α coefficient of the FoP-Q-SF in this study was 0.848.

### 2.5 Statistical analyses

We analyzed data using SPSS 24.0. To enhance data reporting transparency and evaluate the appropriateness of statistical analyses, we examined the distributional characteristics of all continuous variables, including measures of central tendency, dispersion, and distributional shape (i.e., skewness and kurtosis). The results showed that skewness ranged from −0.404 to 0.698 and kurtosis from −0.454 to 0.105, indicating approximate normality (|values| < 1) (Mishra et al., 2019). This finding supported the application of parametric statistical methods (Vickers, 2005). Continuous variables were presented as mean ± standard deviation (M ± SD), while categorical variables were described as frequencies. Pearson’s bivariate correlation analysis was employed to explore the associations among variables, and multicollinearity among variables was assessed using the variance inflation factor (VIF), with a VIF < 5 indicating no significant collinearity issues (Kim, 2019). To test the serial mediation effect, Model 6 of the PROCESS 3.5 macro,^1^ developed by Hayes, was utilized. Statistical significance of indirect effects was tested via bias-corrected bootstrap confidence intervals (CI) with 5,000 resamples. Effects were considered significant if the 95% CI did not include zero. In all analyses, a 2-tailed *p* < 0.05 was considered statistically significant.

## 3 Results

### 3.1 Common method bias test

The results of the Harman’s single-factor test revealed that there were nine factors with eigenvalues exceeding 1, and the explanatory power of the first factor was 33.74%, which was below the critical threshold of 40%. Therefore, the data used in this study do not exhibit any discernible common method bias.

### 3.2 Statistical description and influencing factors of FoP

The socio-demographic and clinical characteristics of the 300 participants included in the study are presented in Tables 1, 2. Overall, 228 male (76.0%) and 72 female (24.0%) patients were included in the sample. The participants’ mean age was 59.52 ± 12.72 years, with 148 participants (49.3%) aged ≥ 60 years.

**Table 1:** Comparison of FoP scores in participants with different socio-demographic characteristics (N = 300).

Socio-demographic characteristics	*N* (%)	FoP scores		
		M ± SD	*t*/*F*	*p*-value
Gender			*t* = −1.579	0.115
Male	228 (76.0)	29.90 ± 7.98		
Female	72 (24.0)	31.72 ± 10.14		
Age group			*F* = 9.474^***^	< 0.001
18–44	36 (12.0)	32.56 ± 7.97		
45–59	116 (38.7)	32.35 ± 8.47		
≥60	148 (49.3)	28.22 ± 8.30		
Education level			*F* = 0.082	0.970
Primary school and below	112 (37.3)	30.14 ± 8.59		
Junior high school	95 (31.7)	30.23 ± 8.69		
Technical secondary school or high school	58 (19.3)	30.79 ± 8.04		
College degree or above	35 (11.7)	30.34 ± 8.56		
Hospitalization costs			*t* = −2.143^*^	0.033
Medical insurance	129 (43.0)	29.12 ± 8.49		
Self-pay	171 (57.0)	31.25 ± 8.53		
Urban–rural distribution			*t* = −3.034^**^	0.003
Urban	159 (53.0)	28.94 ± 8.20		
Rural	141 (47.0)	31.91 ± 8.71		
Employment status			*F* = 18.517^***^	< 0.001
Full-time	140 (46.7)	33.34 ± 9.02		
Retired	78 (26.0)	27.04 ± 6.91		
No job	82 (27.3)	28.35 ± 7.46		
Marital status			*F* = 0.740	0.529
Unmarried	9 (3.0)	29.78 ± 6.10		
Married	256 (85.3)	30.60 ± 8.68		
Divorced	9 (3.0)	30.11 ± 8.88		
Widowed	26 (8.7)	28.00 ± 8.04		
Parental status			*t* = 0.740	0.870
No	10 (3.3)	29.90 ± 5.26		
Yes	290 (96.7)	30.35 ± 8.66		
Family financial situation			*F* = 10.494^***^	< 0.001
Income > Expenditure	36 (12.0)	27.47 ± 7.23		
Income equals expenditure	104 (34.7)	28.18 ± 8.21		
Income < Expenditure	160 (53.3)	32.38 ± 8.58		
Family history of stroke			*F* = 0.530	0.589
No	283 (94.3)	30.28 ± 8.60		
Yes	15 (5.0)	30.60 ± 8.37		
Unclear	2 (0.6)	36.50 ± 4.95		

^***^p < 0.001; ^**^p < 0.01; ^*^p < 0.05.

**Table 2:** Comparison of FoP scores in participants with different clinical characteristics (*N* = 300).

Clinical characteristics	*N* (%)	FoP scores		
		M ± SD	*t*/*F*	*p*-value
Types of stroke			*t* = 3.395^**^	0.001
Hemorrhagic stroke	29 (9.7)	35.38 ± 8.06		
Ischemic stroke	271 (90.3)	29.80 ± 8.45		
Complications			*t* = −1.319	0.188
No	216 (72.0)	29.93 ± 8.06		
Yes	84 (28.0)	31.38 ± 9.70		
Functional impairment			*t* = −5.173^***^	< 0.001
No	209 (69.7)	28.72 ± 8.05		
Yes	91 (30.3)	34.05 ± 8.59		
Other chronic diseases			*F* = 1.625	0.184
No	91 (30.3)	28.92 ± 7.56		
1 kind	109 (36.3)	30.81 ± 8.67		
2 kinds	73 (24.3)	31.68 ± 9.44		
≥3 kinds	27 (9.0)	29.56 ± 8.52		
Daily living activities			*F* = 7.650^***^	< 0.001
Independency	22 (7.3)	28.05 ± 8.37		
Slight dependency	180 (60.0)	29.08 ± 8.13		
Moderate dependency	69 (23.0)	31.84 ± 8.94		
Severe dependency	29 (9.7)	36.28 ± 7.57		
Stroke-related knowledge			*F* = 9.746^***^	< 0.001
Know nothing	152 (50.7)	32.42 ± 8.55		
Know a little	93 (31.0)	28.41 ± 8.11		
Have a fair knowledge	55 (18.3)	27.84 ± 8.03		

^***^p < 0.001; ^**^p < 0.01; ^*^p < 0.05.

Univariable analyses indicated that FoP scores significantly differed across nine variables: age group, hospitalization costs, urban–rural residence, employment status, family financial status, stroke type, functional impairment, activities of daily living, and stroke-related knowledge (*p* < 0.05 for all; see Tables 1, 2).

### 3.3 Statistical description and correlation analysis

The total FoP score in patients with first-ever stroke ranged from 17 to 55 (mean scores: 30.34 ± 8.56). The mean scores of the other variables were as follows: Type D personality (22.16 ± 9.95), PSS (54.06 ± 13.41), and IU (27.82 ± 7.93), as shown in Table 3.

**Table 3:** Statistical description and correlation analysis results (*N* = 300).

Pearson correlation	1	2	3	4
1	–			
2	−0.682^***1)^	–		
3	0.675^***1)^	−0.674^***1)^	–	
4	0.511^***1)^	−0.498^***2)^	0.521^***1)^	–
M ± SD	22.16 ± 9.95	54.06 ± 13.41	27.82 ± 7.93	30.34 ± 8.56

1 = Type D personality; 2 = PSS; 3 = IU; 4 = FoP; ^***^p < 0.001; ^**^p < 0.01; ^*^p < 0.05.

1) Large effect size with a strong correlation; 2) approaching the threshold for a large effect size.

The correlations between variables were investigated using Pearson’s correlation analysis. The Pearson correlation coefficient (*r*) ranges from −1 to +1. Specifically, an absolute value of the Pearson correlation coefficient (|*r*|) of 0.1 reflects a small effect size with a weak correlation, |*r*| = 0.3 reflects a medium effect size with a moderate correlation, and |*r*| = 0.5 reflects a large effect size with a strong correlation (Cohen, 1992). Our results showed that Type D personality was positively correlated with FoP (*r* = 0.511, *p* < 0.001) and IU (*r* = 0.675, *p* < 0.001), and negatively correlated with PSS (*r* = −0.682, *p* < 0.001). Furthermore, PSS was negatively correlated with IU (*r* = −0.674, *p* < 0.001) and FoP (*r* = −0.498, *p* < 0.001). IU was positively correlated with FoP (*r* = 0.521, *p* < 0.001).

### 3.4 Serial mediation model testing

To test the mediating effects of PSS and IU between Type D personality and FoP, we used Model 6 from the PROCESS macro v3.5 developed by Hayes. Several socio-demographic and clinical characteristics significantly associated with FoP in univariate analysis were entered as control variables in the model.

As presented in Table 4, regression analysis showed that Type D personality negatively predicted PSS (*B* = −0.763, *p* < 0.001) and positively predicted IU (*B* = 0.296, *p* < 0.001), while PSS negatively predicted IU (*B* = −0.196, *p* < 0.001). In the full model accounting for all variables, Type D personality maintained a significant direct effect on FoP (*B* = 0.194, *p* = 0.001), and IU positively predicted FoP (*B* = 0.193, *p* = 0.012); however, PSS did not significantly predict FoP (*B* = −0.062, *p* = 0.172). Furthermore, all variables within the regression model had VIFs ranging from 1.103 to 2.376, which suggested the absence of substantial multicollinearity issues.

**Table 4:** Regression analysis of variables within the chain intermediary model.

Outcome variable	Predictor variable	*R^2^*	*F*	*t*	*B*	*β*	*p*-value
PSS	Type D personality	0.528	32.341	−12.239^***^	−0.763	−0.566	<0.001
IU	Type D personality	0.572	34.983	6.845^***^	0.296	0.372	<0.001
	PSS			−5.905^***^	−0.196	−0.331	<0.001
FoP	Type D personality	0.387	15.108	3.212^**^	0.194	0.226	0.001
	PSS			−1.369	−0.062	−0.098	0.172
	IU			2.527^*^	0.193	0.179	0.012

*B* = Unstandardized beta coefficients; *β =* Standardized coefficients; ^***^p < 0.001; ^**^p < 0.01; ^*^p < 0.05.

**Figure 2:**
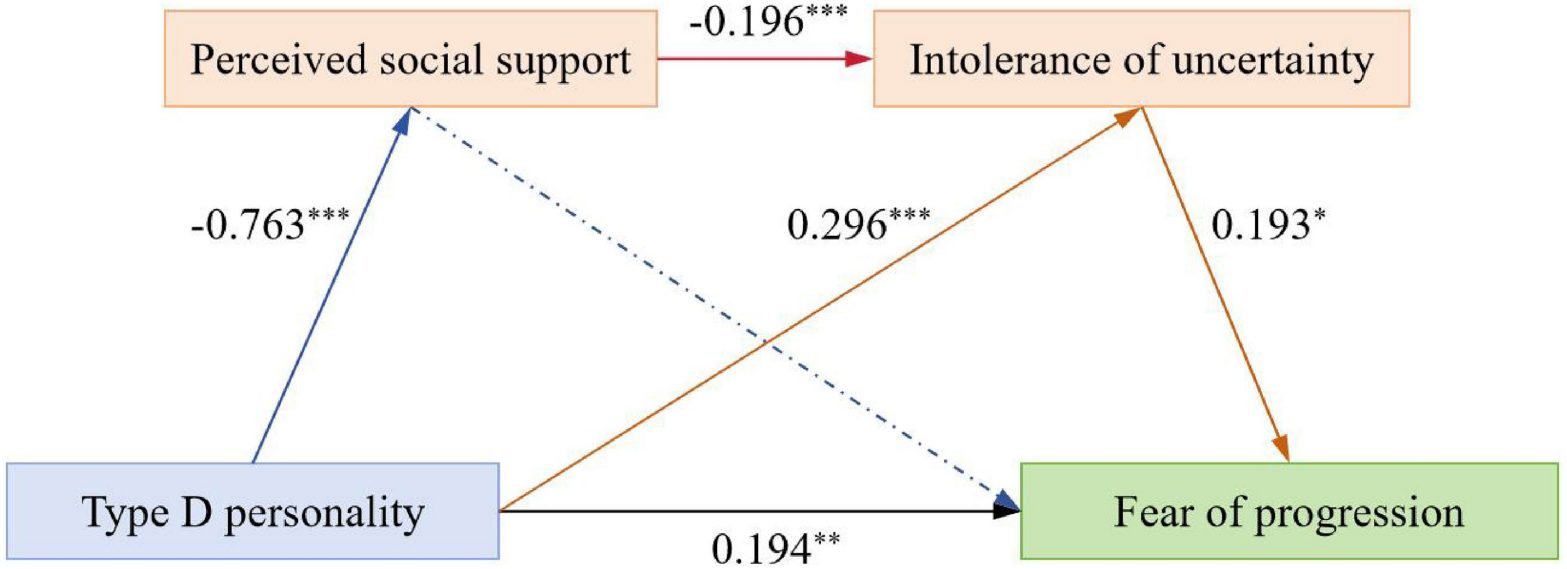
Serial mediation model (***p < 0.001, **p < 0.0.1, and *p < 0.05).

Further mediation analyses are presented in Table 5 and Figure 2. The direct effect of Type D personality on FoP was significant (95% CI [0.072, 0.316]); PSS did not independently mediate the relationship between Type D personality and FoP (95% CI [−0.023, 0.140]); however, IU significantly mediated the Type D personality-FoP relationship (95% CI [0.005, 0.111]). Furthermore, PSS and IU had a significant serial mediation role in the relationship between Type D personality and FoP (95% CI [0.002, 0.066]), accounting for 8.84% of the total effect.

**Table 5:** Multiple mediating models between Type D personality and FoP.

Pathways	Effect size	*Se*	Bootstrap 95% CI	Relative effect (%)
Ind1	0.047	0.042	−0.023 to 0.140	14.33
Ind2	0.057	0.027	0.005–0.111	17.38
Ind3	0.029	0.016	0.002–0.066	8.84
Total	0.328	0.046	0.237–0.419	100
Direct effect	0.194	0.060	0.075–0.313	59.15
Total indirect effect	0.133	0.047	0.049–0.230	40.55

Ind1: Type D personality → PSS → FoP; Ind2: Type D personality → IU → FoP; Ind3: Type D personality → PSS → IU → FoP.

## 4 Discussion

### 4.1 FoP in first-ever stroke patients

In this study, the mean score of FoP among first-ever stroke patients (30.34 ± 8.56) was lower than that reported by Guan et al. (2024). This divergence may be attributed to differences in stroke subtype composition. In this study, 90.3% of the participants had ischemic stroke while 9.7% had hemorrhagic stroke, whereas patients with hemorrhagic stroke comprised 35.4% of Guan et al. (2024)’s sample. As hemorrhagic stroke is clinically associated with higher acute mortality (Lim et al., 2024), patients with this subtype typically demonstrate heightened FoP, potentially explaining the elevated scores in Guan et al. (2024)’s study. Notably, the scores of patients with first-ever stroke on the two subscales—Physical Health and Social-Family—were essentially equivalent, demonstrating that the occurrence and progression of stroke impose comparably significant impacts on patients’ physical well-being and their family and social domains. Therefore, neurologists and nurses should implement routine FoP screening and deliver targeted interventions—including disease education and coping skills training—to mitigate FoP in high-risk first-ever stroke patients.

### 4.2 Type D personality in first-ever stroke patients

In this study, the prevalence of Type D personality in patients with first-ever stroke was 37.3%. This substantially exceeded the prevalence rate of 22.2% reported in the general population (Beutel et al., 2012). The mean Type D personality score in this study was 20.22 ± 8.69, which is similar to the score reported by Yin et al. (2023). Yao et al. (2022) conducted a study to examine the association between Type D personality and ischemic cerebrovascular disease; the results showed that Type D individuals had a higher frequency of acute ischemic stroke and white matter hyperintensity, which further elucidated the mechanism of Type D personality predisposing to stroke from the perspective of imaging. Our results also confirmed the high incidence of Type D personality in patients with stroke. Consequently, we propose that the DS-14 is a brief, well-validated measure of Type D personality, which can be incorporated into clinical research and practice to facilitate the identification of high-risk stroke patients by neurologists and nurses. For patients at high risk of Type D personality, interventions targeting enhanced social support and cognitive behavioral therapy should be considered (Purdy, 2013; Su and He, 2019).

### 4.3 Type D personality positively predicted FoP

Consistent with our first hypothesis, the results of this study indicated that Type D personality positively predicted FoP in patients with first-ever stroke. This aligns with the findings of previous research (Wu et al., 2025). Patients with stroke exhibiting Type D personality are more prone to being sensitive to the pain and burden of the disease. Heightened sensitivity increases their awareness of the disease threat, fostering excessive pessimism regarding prognosis. This pessimism, in turn, generates substantial concerns about treatment efficacy, financial issues, and future life. Moreover, due to deficits in emotional regulation skills and a lack of social support, these patients cannot effectively alleviate their negative emotions. As a result, they persist in a state of chronic stress characterized by FoP.

### 4.4 The serial mediation role of PSS and IU

The serial mediation model demonstrated a non-significant independent mediating effect of PSS between Type D personality and FoP, contradicting Hypothesis 2. However, IU maintained a significant independent mediating effect (Hypothesis 3 was supported). This demonstrates that the role of IU takes precedence over PSS’ mediating role when both are modeled concurrently, establishing IU as the primary mediator linking Type D personality to FoP.

Our study further confirmed the serial mediating role of PSS and IU in the pathway linking Type D personality to FoP among first-ever stroke patients, thus supporting Hypothesis 4. This finding aligns with the theoretical propositions of the Stress and Coping Theory, demonstrating a sequential pathway from the stressor (stroke event) through compromised coping resources (lower PSS resulting from type D personality) to threat appraisal (IU), culminating in the stress response (FoP). Specifically, Type D personality impairs patients’ capacity to proactively leverage social support. Given stroke severity and interindividual variability, even experienced clinicians cannot reliably predict disease progression. This prognostic uncertainty poses a significant challenge for stroke patients. However, diminished PSS predicts inadequate resources for coping with uncertain events in patients with stroke (Yu et al., 2022) and reinforces a persistent negative belief that disease-related uncertainty is intolerable and unacceptable (Malivoire et al., 2019), thereby exacerbating stroke survivors’ fears of disease deterioration or recurrence (Shen et al., 2024).

Our findings indicate that neurologists and nurses could mitigate FoP among patients with type D personality through enhancing of PSS or improvement of patients’ tolerance of uncertainty. Empirical studies demonstrate that nurse-led peer support interventions effectively enhance PSS in stroke survivors (Wan et al., 2024). Tai Chi and meaningful life interventions have also been shown to significantly boost PSS levels (Koren et al., 2021; Liu et al., 2024). Additionally, cognitive behavioral therapy can reduce the IU level by regulating disease perception and cognitive-emotional coping styles (Talkovsky and Norton, 2016).

## 5 Strengths and limitations

Our research confirms that PSS and IU exert a serial mediating effect on the relationship between Type D personality and FoP. This finding provides a theoretical basis and empirical support for developing interventions to mitigate FoP in patients with stroke, thereby extending previous research in this field. Despite this study’s contributions, certain limitations must be acknowledged. The use of a cross-sectional design constrains causal inference regarding the relationships among the variables. Moreover, convenience sampling was employed from two hospitals, with all participants being stroke survivors from the same region in China. This sampling method inevitably introduced selection bias, limiting the generalizability of this study’s findings. Finally, the results remain unvalidated in other stroke populations, particularly among recurrent stroke survivors.

Considering these limitations, future research should focus on: (1) Longitudinal studies to establish causal relationships or design intervention trials targeting enhanced PSS. (2) A multicenter investigation should be conducted, enrolling patients with stroke with diverse characteristics (e.g., recurrent stroke, comorbid dementia, or depression), to validate the model’s effectiveness across heterogeneous populations.

## 6 Conclusion

Type D personality significantly predicts FoP in first-ever stroke patients. Although such traits are generally stable and intervention—resistant, our serial mediation model demonstrates that while PSS alone does not mediate the Type D—FoP relationship, the PSS—IU serial pathway is significant. This implies enhancing PSS may reduce FoP by alleviating IU. These findings indicate that interventions targeting enhanced PSS and reduced IU represent a promising approach to alleviate FoP in these patients, despite the inherent stability of Type D personality traits.

## Data Availability

The original contributions presented in the study are included in the article/supplementary material, further inquiries can be directed to the corresponding authors.

## Footnotes

^1^ http://www.afhayes.com
